# Free Water as a Marker of Early Small Vessel Disease in Healthy Aging

**DOI:** 10.1111/ene.70094

**Published:** 2025-03-04

**Authors:** Manuel Leitner, Lukas Pirpamer, Edith Hofer, Stefan Ropele, Ofer Pasternak, Reinhold Schmidt, Marisa Koini

**Affiliations:** ^1^ Department of Neurology Medical University of Graz Graz Austria; ^2^ Medical Image Analysis Center (MIAC) and Department of Biomedical Engineering University of Basel Basel Switzerland; ^3^ Institute for Medical Informatics, Statistics and Documentation Medical University of Graz Graz Austria; ^4^ Department of Psychiatry, Brigham and Women's Hospital Harvard Medical School Boston Massachusetts USA; ^5^ Department of Radiology, Brigham and Women's Hospital Harvard Medical School Boston Massachusetts USA

**Keywords:** diffusion tensor imaging, free water, healthy aging, marker, small vessel disease

## Abstract

**Background:**

Brain aging is associated with cerebrovascular changes and related microstructural pathology. In this context, research indicates that small vessel disease (SVD) is characterized by increases in extracellular free water (FW).

**Methods:**

We examined 94 individuals with early signs of small vessel disease (eSVD; *M*_*age*_ = 69.47 years, SD_*age*_ = 8.27; 59.6% female) and 94 age‐matched controls (CON; *M*_*age*_ = 70.80 years, SD_*age*_ = 8.59, 59.6% female). Individuals with eSVD were defined by having a white matter hyperintensity (WMH) score ≥ 2 or showing the presence of lacunes or microbleeds. We examined differences in the diffusion MRI measures between both groups. In addition, we assessed the association between age and the diffusion MRI measures averaged across the entire normal‐appearing white matter (NAWM). To test if FW mediates the association of FW‐uncorrected DTI measures and age, we applied simple mediation models.

**Results:**

Differences between individuals with eSVD and CON were identified for FW in NAWM and FW‐uncorrected DTI, but not for FW‐corrected DTI measures. Both FW and FW‐uncorrected DTI measures in NAWM were significantly associated with age across the total sample (*r*_*range*_ = −0.24 to 0.44) and across each group (*r*_*range*_ = −0.36 to 0.48). In addition, the association between FW‐uncorrected DTI measures and age was mediated by FW.

**Conclusions:**

Even in subjects with subtle, clinically silent, cerebral white matter changes, increases in FW could be observed compared to individuals without those changes. Therefore, FW might act as a sensitive marker at the earliest signs of SVD.

## 1 Introduction

Aging is associated with an increasing prevalence of cerebral small vessel disease (SVD) [1], a medical condition that impacts arterioles, capillaries, and small veins within the brain [2]. On a macroscopic scale, these changes are visible on magnetic resonance imaging (MRI) as white matter hyperintensities (WMHs), lacunes, microbleeds, microinfarcts, enlarged perivascular spaces, and brain atrophy [3, 4].

Diffusion MRI is a technique that allows detecting microstructural alterations of brain tissue that are common in SVD by quantifying the diffusion properties of water molecules [5, 6]. Diffusion Tensor Imaging (DTI) techniques provide information about the microstructure of the white matter in the brain [5]. However, as the interpretation of DTI metrics depends on the local fiber architecture and thus requires careful interpretation, the precise neurobiological foundations cannot be directly inferred [7]. One approach to compensate for these limitations in DTI and contamination of diffusion‐weighted metrics caused by, for instance, extracellular free water (FW) [8], is the use of a FW‐corrected DTI model [9, 10].

The FW diffusion MRI model is used to distinguish between a FW compartment, representing water molecules that are free to diffuse in any direction, and the tissue compartment, comprising water molecules with diffusion that is hindered or restricted. The tissue compartment represents intra‐ and extracellular water molecules affected by physical barriers, for instance, myelin or axonal membranes, and thus reflects the cellular organization of white matter [11]. This compartment is modeled by a diffusion tensor from which conventional DTI parameters, such as fractional anisotropy (FA), mean diffusivity (MD), axial diffusivity (AD) and radial diffusivity (RD) can be derived as FW‐corrected DTI parameters. By eliminating the FW portion from diffusion properties of brain tissue, not only do the metrics appear more accurate, but also the sensitivity of DTI parameters to age‐related alterations in the white matter increases [12, 13, 14]. Free‐water elimination (FWE) has previously been used to demonstrate the relevance of increased FW levels for age‐related changes in white matter separate from tissue modifications [15, 16]. Archer and colleagues (2020) could illustrate that FW was associated with cognitive functioning, surpassing the influence of volume reduction (e.g., in the hippocampal area), indicating a unique role of FW in cognitive decline [15]. Additionally, FW in normal‐appearing white matter (NAWM) was found to be negatively correlated with neuropsychological measures such as executive function [17]. Therefore, studies suggest incorporating FWE in the investigation of brain aging, as FW could potentially serve as a valuable marker for age‐related brain changes and cognitive deterioration [16, 17].

The association of aging and FW‐uncorrected DTI parameters has been demonstrated multiple times in the past, showing decreases in FA, increases in MD, and inconsistent results for AD and RD [18, 19, 20, 21, 22, 23]. However, the relationship between age and DTI measures was shown to be less pronounced after applying FWE [14]. Even though SVD manifests in both focal and widespread microstructural tissue changes, previous research showed that diffusion alterations in SVD are mostly driven by the amount of FW rather than alterations of the white matter fiber organization [11]. The authors could further demonstrate a reduction in global white matter FA as well as an increase in MD and FW when comparing individuals with genetically defined SVD to healthy controls (CON). FW could therefore act as a possible marker to detect cerebral SVD degeneration and an associated cognitive decline [24]. A more in‐depth investigation of the role of FW in the interaction with age and early signs of SVD is thus mandatory to examine its sensitivity as a marker in a pre‐clinical stage of SVD.

The aim of the present study is (1) to determine whether FW and DTI metrics differentiate pre‐clinical SVD from unremarkable cerebral macrostructure by comparing individuals with and without early cerebral pathology, (2) to examine the association of FW and DTI metrics with aging in people with and without early signs of SVD, and (3) to assess whether FW acts as a mediator between age and uncorrected DTI metrics in order to inspect *how* age exerts its effect on alterations in these MRI measures.

We hypothesize that (1) FW qualifies as a potential marker to differentiate between pre‐clinical SVD from unremarkable cerebral macrostructure, (2a) FW is associated with aging in pre‐clinical SVD but also in subjects with normal cerebral macrostructure, (2b) the association between FW‐uncorrected DTI parameters and age will decrease upon correction for FW, and (2c) there exists a group difference in the association of age and DTI metrics. We further hypothesize that (3) FW acts as a mediator between age and uncorrected DTI metrics.

## 2 Material and Methods

### 2.1 Participants

The Austrian Stroke Prevention Family Study (ASPS‐Fam) is an extension of the single‐centre and community‐dwelling Austrian Stroke Prevention Study (ASPS). The ASPS was established in 1991 in order to investigate the neurological effects of vascular risk factors in the normal adult population of Graz, Austria [25, 26]. From 2006 to 2013, study participants of the ASPS and their first‐degree relatives were recruited for the ASPS‐Fam. The inclusion criteria consisted of a normal neurological examination and no personal history of prior stroke or dementia. Overall, 419 individuals from the ASPS‐Fam underwent MRI, with 381 of them belonging to 169 different families. The number of family members included varied from 2 to 6. Further, all participants underwent a comprehensive diagnostic examination, including clinical history, laboratory examination, neuropsychological testing, and an extensive evaluation of vascular risk factors. A total of 267 individuals had a diffusion‐weighted MR scan, and 237 successfully passed the data quality assessment.

The individuals were categorized into two groups according to their brain health: Healthy (i.e., the control group, CON) was determined based on a WMH score of 0 or 1 [27], and the absence of lacunes and microbleeds. The presence of a WMH score ≥ 2, lacunes, and/or microbleeds was considered early signs of SVD (eSVD). Lacunes were defined as focal lesions in the basal ganglia, internal capsule, thalamus, or brainstem that did not exceed a diameter of 15 mm. As the healthy group was significantly younger compared to individuals with early signs of SVD, the groups were matched according to age. Therefore, propensity score matching was employed using the SPSS software and a common threshold (caliper) of 0.10. This led to a final sample of 188 age‐matched subjects aged between 45 and 87 years (*M* = 70.14, SD = 8.44) examined in this study.

### 2.2 MRI Data Acquisition

A 3T whole‐body MR system (TimTrio; Siemens Healthcare, Erlangen, Germany) with a 12‐channel head coil was used to acquire MRI data. For DTI, a 2D echo planar imaging (EPI) diffusion sequence with 12 directions, *b*‐value = 1000 s/mm^2^, TR/TE = 6700/95 ms, resolution = 2 × 2 × 2.5 mm, matrix = 128 × 128 px, number of slices = 50, parallel imaging (GRAPPA = 2), with one b0 image and three repetitions was used. For tissue segmentation, a high‐resolution T1 weighted 3D sequence with magnetization prepared rapid acquisition of gradient echo (MPRAGE) with whole brain coverage (TR = 1900 ms, TE = 2.19 ms, TI = 900 ms, flip angle = 9°, isotropic resolution of 1 mm) was included in the study protocol. Further, a T2w‐FLAIR sequence (TR = 10,000 ms, TE = 69 ms, TI = 2500 ms, number of slices = 44, slice thickness = 3 mm, in‐plane resolution = 0.86 × 0.86 mm^2^) was used to assess white matter lesion load.

### 2.3 Preprocessing and Image Analysis

The Functional Magnetic Resonance Imaging of the Brain (FMRIB) Diffusion Toolbox (FDT [28]) which is included in the FMRIB Software Library (FSL, version 6.0.1 [29]) was used to process diffusion data. First, a brain mask in the b0 image was extracted for each participant with FSL‐bet, followed by motion correction and eddy‐current correction using FSL‐eddy [30].

After visual inspection of the data, FW‐corrected and uncorrected diffusion tensors for each voxel were calculated [9]. In short, the diffusion signal of each voxel was fitted to a two‐compartment model. The tissue compartment was modeled by a diffusion tensor, and the FW compartment was modeled as an isotropic diffusion tensor with a fixed diffusivity reflecting free diffusion at 37°C. The FW value represents the relative contribution of the FW compartment in each voxel, ranging from 0 to 1. The tissue compartment measures (which are denoted by the suffix [‐.t] for tissue) are derived from the FW corrected diffusion tensor and include fractional anisotropy (FA.t), axial diffusivity (AD.t), mean diffusivity (MD.t) and radial diffusivity (RD.t).

To estimate the volume of WMH, binary masks were drawn on the T2w‐FLAIR sequence by an experienced rater. WMH maps were generated by using a threshold‐based seed‐growing algorithm as described previously [31]. Thereby, the rater identified each lesion slice by slice by setting a seed inside each lesion, and the algorithm automatically drew the borders of the lesion. In case of misclassification, the rater modified the lesion masks manually. WMH volume was obtained by fslstats (part of FSL) and evaluated in the native FLAIR space. WM masks were obtained by regional brain segmentation on the T1‐weighted MPRAGE sequence, generated by the FreeSurfer image analysis suite (v 6.0).

To assess the regional median‐DTI measures, the T1 and FLAIR sequences were registered to the diffusion space, and the transformation matrices were applied to the binary WM and WMH masks. The registration was done in FSL‐flirt with six degrees of freedom and a mutual‐information cost function [32]. Besides manually inspecting each registration, we applied a slight erosion of the WM mask to avoid partial volume effects using a threshold of 90% after trilinear interpolation of the WM‐mask, registered into the diffusion space. By subtracting the WMH masks from the FreeSurfer‐segmented WM masks, an individual normal‐appearing white matter (NAWM) mask was computed for each participant. The assessment of median values of FW‐corrected FA.t, AD.t, MD.t, and RD.t was carried out using FSL‐stats.

### 2.4 Statistical Analyses

The statistical analyses were performed using the software SPSS (version 29 [33]) including the Hayes SPSS PROCESS Macro (version 4.3 [34]) (http://www.processmacro.org/download.html). Scatterplots and bar charts were generated with R Studio [35]. Statistical significance was determined using a two‐sided *p*‐value threshold of < 0.05. The following imaging variables in NAWM were included in the analyses: FW, FA, AD, MD, RD, FA.t, AD.t, MD.t, and RD.t (.t‐suffix: corrected for FW).

*T*‐tests were performed in order to determine differences in FW, FW uncorrected, and FW‐corrected DTI measures between individuals with eSVD and CON (Hypothesis 1). To obtain more robust results in the presence of potential violations of assumptions, bootstrapping was performed by using 10.000 bootstrap samples and bias‐corrected and accelerated (BCa) confidence intervals. Pearson correlations were performed to assess the association of the diffusion MRI parameters and age (Hypothesis 2a, 2b). Fisher's r‐to‐z transformations were used to examine differences in correlations between individuals with eSVD and CON (Hypothesis 2c). To adjust for multiple testing, a correction controlling for the false discovery rate (FDR) was applied [36]. To examine if FW in the NAWM mediates the relationship between age and FW‐uncorrected DTI parameters, mediation analyses in the total sample and the two subgroups were performed (Hypothesis 3). 95% confidence intervals were estimated using a bootstrap‐based method developed by Preacher and Hayes [37]. If the 95% percentile bootstrap confidence interval of the indirect effect does not contain 0, a significant mediation effect was assumed. Mediation analyses were performed with the PROCESS macro implemented in SPSS.

## 3 Results

Overall, 188 age‐matched individuals were included in the analyses: 94 individuals who were considered healthy controls (age: *M* = 69.47, SD = 8.27; 59.6% female) and 94 individuals who were healthy but considered to have eSVD including WMHs (grade 2 and 3), lacunes, and/or microbleeds (age: *M* = 70.80, SD = 8.59; 59.6% female). A summary of the demographic and clinical characteristics for each group is presented in Table 1. 75 individuals (39.9%) showed a WMH score of ≥ 2, 23 individuals (12.2%) showed lacunes, and 16 individuals (8.5%) had microbleeds. Overall, in 3 individuals (1.6%), all three signs of brain pathology were present. Our results indicate that individuals with early signs of small vessel disease (MR = 124.57) exhibit significantly greater WMH volume compared to our control group (MR = 64.43) (*U* = 1591.00, *Z* = −7.58, *p* < 0.001, *r* = −0.55).

**TABLE 1:** Differences in demographic and clinical characteristics between healthy elderly (CON) and those with early signs of small vessel disease (eSVD).

Variable	CON (*n* = 94)	eSVD (*n* = 94)	Significance
Age, mean (SD)	69.47 (8.27)	70.80 (8.59)	*p* = 0.281
Sex (% female)	56 (59.6)	56 (59.6)	*p* > 0.999
Education, *n* (%)			***p* = 0.022**
Primary school	14 (14.9)^a^	20 (21.3)^a^	
Apprenticeship	40 (42.6)^a^	41 (43.6)^a^	
High school	20 (21.3)^a^	27 (28.7)^a^	
University/college	20 (21.3)^a^	6 (6.4)^b^	***p* = 0.003**
Hypercholesterolemia, *n* (%)	76 (80.9)	71 (75.5)	*p =* 0.377
Hypertension, *n* (%)	59 (62.8)	67 (71.3)	*p =* 0.215
Alcohol, *n* (%)	58 (61.7)	54 (57.4)	*p* = 0.552
Current smoker, *n* (%)	9 (9.8)	7 (7.4)	*p =* 0.570
Diabetes, *n* (%)	12 (12.8)	12 (12.8)	*p* = 1.000

*Note:* Values represent means and standard deviations (for continuous variables) or frequencies with corresponding percentages (for categorical variables). For Chi‐square tests, the same subscript letters (e.g., a and a) denote a subset of group categories whose column proportions do not differ significantly from each other, while different letters (e.g., a and b) indicate that the column proportions significantly differ from each other at the 0.05 level. Significant *p*‐values are highlighted in bold. For current smoker: *n* = 186.

Abbreviations: CON, elderly controls; eSVD, individuals with early signs of small vessel disease.

**FIGURE 1:**
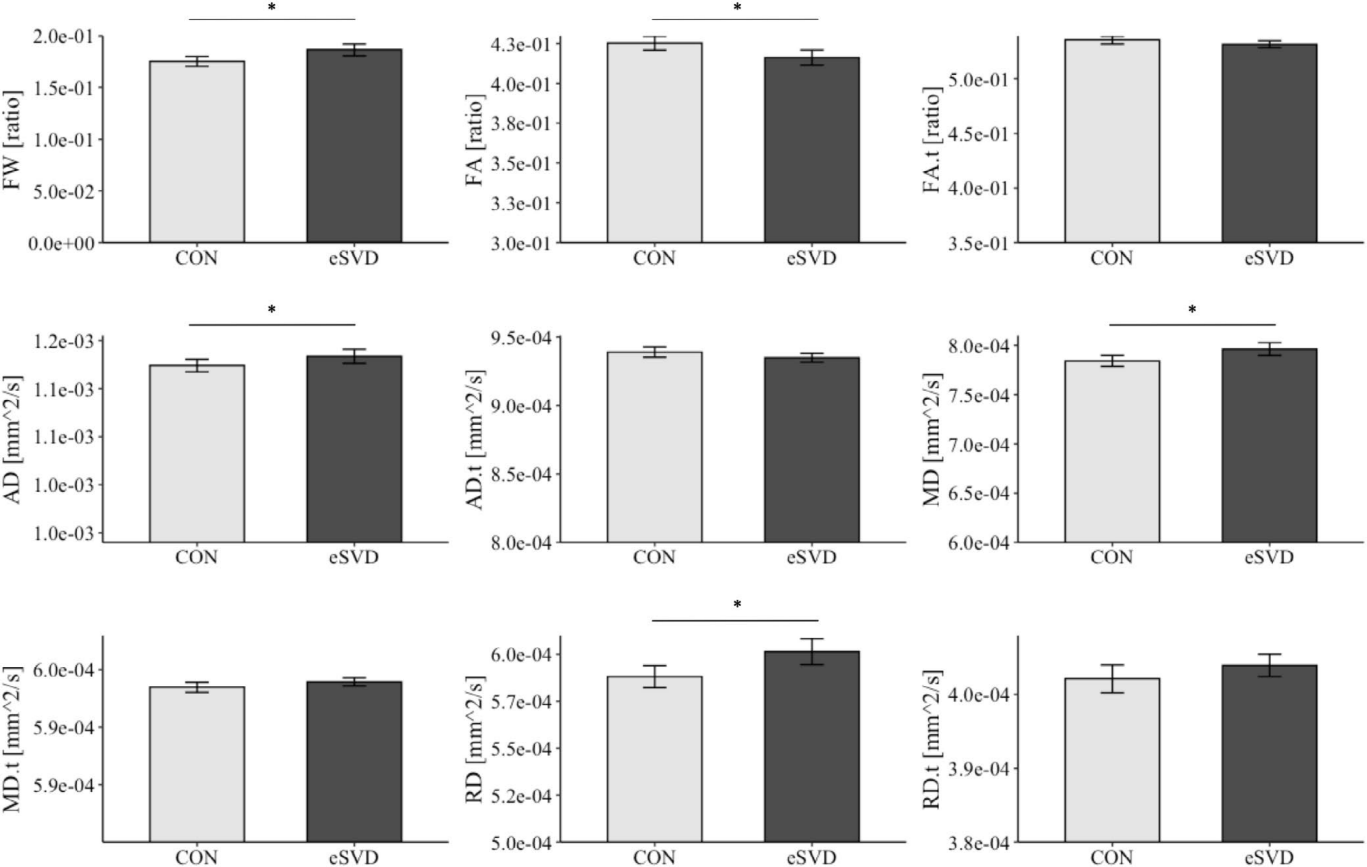
Differences in DTI measures between CON and individuals with eSVD. Graphs marked with an asterisk (*) indicate a significant group difference based on *t‐*tests performed with 95% bias‐corrected and accelerated (BCa) bootstrap confidence intervals (10.000 bootstrap samples). ‐.t (suffix) = corrected for free water; AD, axial diffusivity; CON, elderly controls; eSVD, individuals with early signs of small vessel disease; FA, fractional anisotropy; FW, free water; MD, mean diffusivity; RD, radial diffusivity.

The group comparisons regarding FW, FW‐uncorrected, and FW‐corrected DTI parameters revealed that individuals with eSVD exhibited higher levels of FW, lower levels of FA, as well as higher levels of AD, MD, and RD compared to CON (Figure 1, Table 2).

**TABLE 2:** *T*‐test results for the difference in DTI parameters and FW between CON and individuals with eSVD.

DTI	CON		eSVD		Mean difference [BCa 95% CI]
	*M*	SD	*M*	SD	
FW	1.75e‐1	2.37e‐2	**1.86e‐1**	2.82e‐2	**‐1.11e‐2 [−1.85e‐2, −3.74e‐3]**
FA	**4.25e‐1**	2.25 e‐2	4.16e‐1	2.30e‐2	**9.11e‐3 [2.60e‐3, 1.56e‐2]**
AD	1.17e‐3	3.25e‐5	**1.18e‐3**	3.68e‐5	**−9.54e‐6 [−1.93e‐5, −1.19e‐7]**
MD	7.84e‐4	2.75e‐5	**7.96e‐4**	3.24e‐5	**−1.20e‐5 [−2.04e‐5, −3.58e‐6]**
RD	5.88e‐4	2.93e‐5	**6.01e‐4**	3.39e‐5	**−1.33e‐5 [−2.20e‐5, −4.34e‐6]**
FA.t	5.36e‐1	1.84e−2	5.32e−1	1.55e‐2	4.04e‐3 [−8.86e‐4, 8.79e‐3]
AD.t	9.39e‐4	1.85e‐5	9.34e‐4	1.58e‐5	4.11e‐6 [−9.22e‐7, 8.87e‐6]
MD.t	5.97e‐4	1.08e‐6	5.97e‐4	8.65e‐7	‐2.31e‐7 [−5.09e‐7, 4.4e‐8]
RD.t	4.02e‐4	9.35e‐6	4.04e‐4	7.51e‐6	‐1.82e‐6 [−4.20e‐6, 5.98e‐7]

*Note:* Bias corrected and accelerated (BCa) 95% bootstrap confidence intervals (CI) are reported in brackets. Confidence intervals are based on 10.000 bootstrap samples. Significant results are highlighted in bold (CI not containing 0).

Abbreviations: ‐.t (suffix), corrected for free water; AD, axial diffusivity; CON, elderly controls; eSVD, individuals with early signs of small vessel disease; FA, fractional anisotropy; FW, free water; *M*, mean; MD, mean diffusivity; RD, radial diffusivity; SD, standard deviation.

**FIGURE 2:**
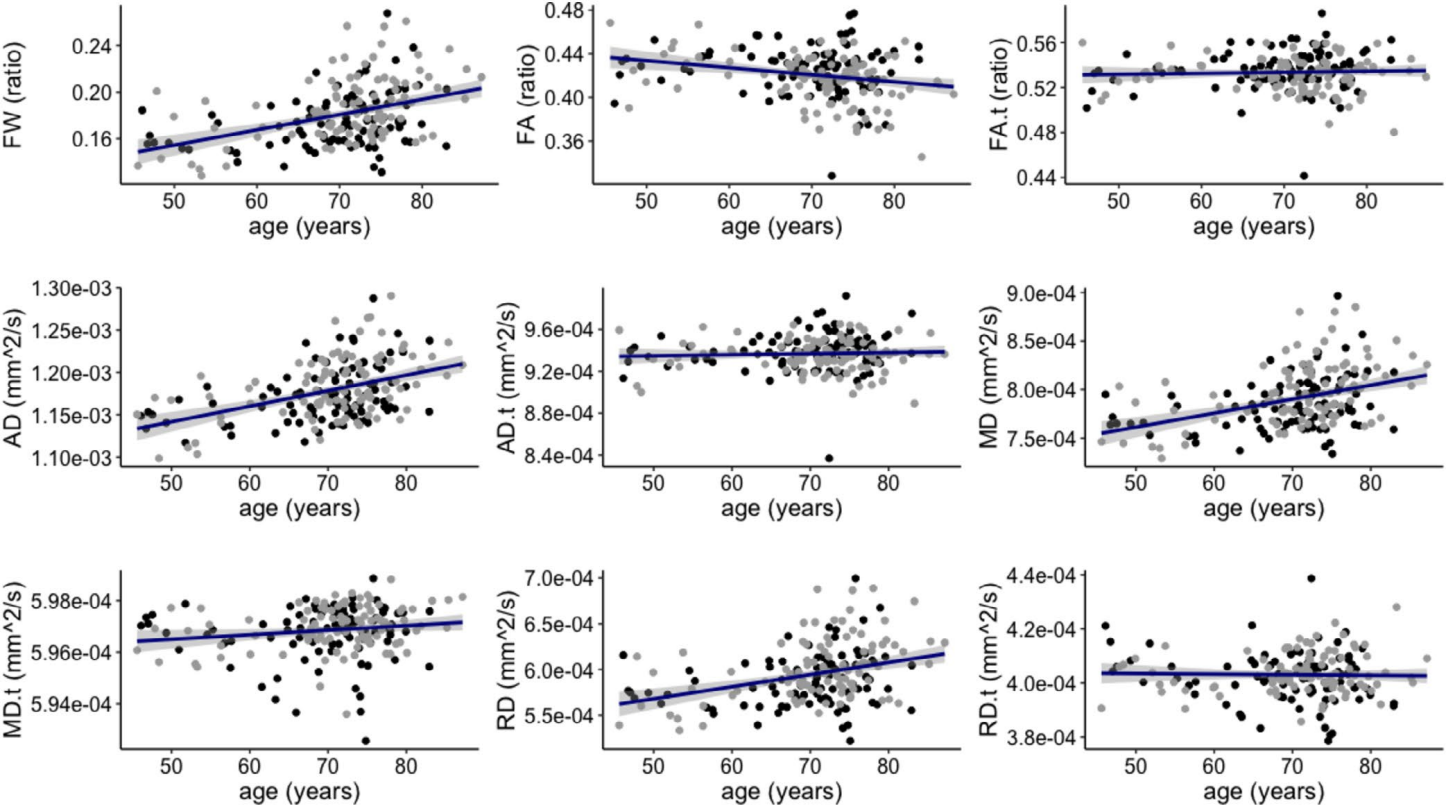
Association between FW and DTI parameters in NAWM with age. The gray dots represent individuals with eSVD, and the black dots represent the control group (CON). The dark blue line represents a trend line for the total sample (*n* = 188) with the shading around the regression line representing the 95% confidence interval. ‐.t (suffix), corrected for free water; AD, axial diffusivity; FA, fractional anisotropy; FW, free water; MD, mean diffusivity; RD, radial diffusivity. 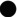
CON, 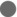
eSVD.

Pearson correlation analyses (*p*‐values corrected for multiple testing) in the entire sample revealed that FW in NAWM and FW‐uncorrected DTI parameters (FA, AD, MD, RD) were all significantly correlated with age. However, this correlation was not significant for the FW‐corrected DTI parameters (Table 3, Figure 2). Similar results were also found in the subgroups of CON and individuals with eSVD, as shown in Table 3. None of the correlations showed significant differences between CON and patients with eSVD after applying FDR correction.

**TABLE 3:** Pearson correlations for FW, FW‐uncorrected, and FW‐corrected DTI parameters with age in the total sample and both subgroups.

	Total (*n* = 188)		CON (*n* = 94)		eSVD (*n* = 94)		Differences in *r*	
	*r*	*p*	*r*	*p*	*r*	*p*	*z*	*p*
FW × age	0.42	**< 0.001**	0.33	0.**001**	0.48	**< 0.001**	−1.22	0.111
FA × age	−0.24	0.**001**	−0.08	0.435	−0.36	**< 0.001**	2.01	0.022^ns^
AD × age	0.44	**< 0.001**	0.41	**< 0.001**	0.47	**< 0.001**	−0.49	0.311
MD × age	0.40	**< 0.001**	0.30	0.**003**	0.47	**< 0.001**	−1.29	0.099
RD × age	0.35	**< 0.001**	0.22	0.037^ns^	0.44	**< 0.001**	−1.70	0.045^ns^
FA.t × age	0.05	0.536	0.17	0.112	−0.07	0.510	1.59	0.056
AD.t × age	0.05	0.486	0.13	0.205	−0.02	0.862	1.017	0.155
MD.t × age	0.15	0.038^ns^	0.04	0.718	0.27	0.**008**	−1.64	0.050
RD.t × age	−0.02	0.768	−0.19	0.067	0.16	0.124	−2.39	0.009^ns^

*Note:* The original *p*‐values represented in the table are not FDR corrected. However, a subscript ns indicates that the correlation did not reach significance after the FDR correction was applied. Also, the results of a Fisher's *r*‐to‐*z* transformation to compare the differences in *r* between the individuals with early signs of small vessel disease (eSVD) and elderly controls (CON) are presented. Significant *p*‐values are highlighted in bold.

Abbreviations: ‐.t (suffix), corrected for free water; AD, axial diffusivity; FA, fractional anisotropy; FW, free water; MD, mean diffusivity; RD, radial diffusivity.

**FIGURE 3:**
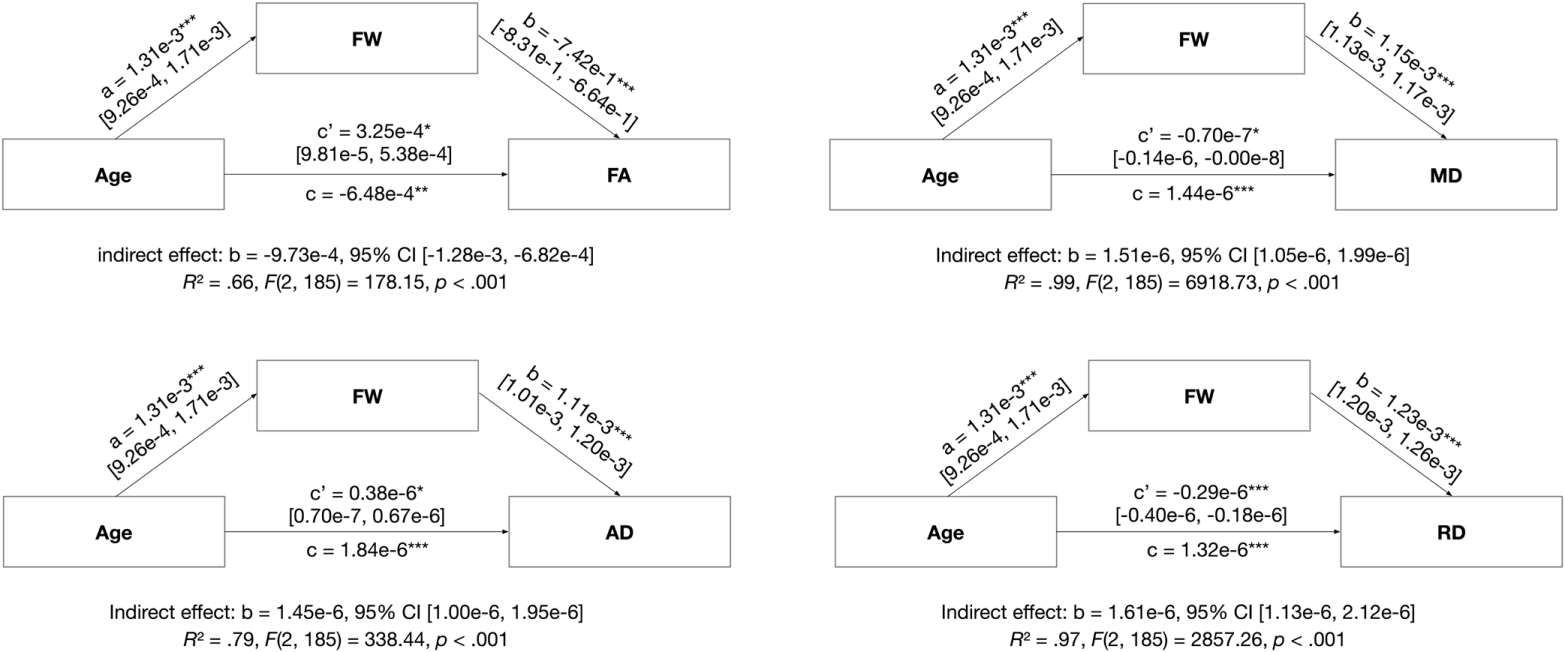
Results of the mediation analyses in the total sample highlighting the path estimates of the independent variable (i.e., age) on the dependent variables (i.e., FW‐uncorrected DTI parameters) including FW as a mediator. a*b = indirect effect, c’ = direct effect, c = total effect. Confidence intervals represent 95% percentile bootstrap intervals (10.000 bootstrap samples). *p < 0.05, **p < 0.01, ***p < 0.001. AD, axial diffusivity; FA, fractional anisotropy; FW, free water; MD, mean diffusivity; RD, radial diffusivity.

Mediation analyses in the total sample revealed that the association between age and the FW‐uncorrected DTI parameters FA, AD, MD, and RD is mediated by FW in NAWM. The same pattern was present in both CON, as well as in individuals with eSVD (Table 4). The results of the mediation analyses for the entire sample are depicted graphically in Figure 3.

**TABLE 4:** Mediating effect of FW on the association between age and FW‐uncorrected DTI measures (FA, MD, AD, RD).

Group	DTI‐parameter	Indirect effect: a × b [95% bootstrap CI]	*R* ^2^	*p*
eSVD	FA	*b* = −1.10e‐3 [−1.51e‐3, −0.68e‐3]	0.71	**< 0.001**
	MD	*b* = 1.80e‐6 [1.09e‐6, 2.50e‐6]	0.99	**< 0.001**
	AD	*b* = 1.79e‐6 [1.06e‐6, 2.59e‐6]	0.81	**< 0.001**
	RD	*b* = 1.89e‐6 [1.15e‐6, 2.64e‐6]	0.97	**< 0.001**
CON	FA	*b* = −7.19e‐4 [−1.14e‐3, −0.35e‐3]	0.59	**< 0.001**
	MD	*b* = 1.09e‐6 [0.55e‐6, 1.72e‐6]	0.99	**< 0.001**
	AD	*b* = 1.05e‐6 [0.50e‐6, 1.69e‐6]	0.76	**< 0.001**
	RD	*b* = 1.18e‐6 [0.58e‐6, 1.83e‐6]	0.96	**< 0.001**

*Note:* Results are listed separately for individuals with early signs of small vessel disease (eSVD) and elderly controls (CON). All FW‐uncorrected DTI measures (FA, MD, AD, RD) were significantly predicted by both age (predictor) and free water (mediator). The explained variance of the DTI measures is listed in the *R* ^2^ column. Significant *p*‐values are highlighted in bold.

## 4 Discussion

In this study, we were able to demonstrate that individuals with early signs of small vessel disease (eSVD) showed higher levels of FW as compared to a clinically healthy, community‐dwelling elderly sample (CON). We further found that FW is correlated with aging irrespective of an early small vessel pathology, and we identified that FW mediates the association between increasing age and changes in FW‐uncorrected DTI parameters, as the correlation with age diminishes when FW is included as a mediator in the model.

Up to now, several studies have already examined the value of FW‐corrected DTI parameters and FW in diseases such as SVD [11, 38], Alzheimer's disease [39], Parkinson's disease [40], traumatic brain injury [41], and multiple sclerosis [42]. These studies confirm that FW can be used to detect cerebral pathologies that are characteristic of several neurological diseases. This aligns with our results, as the significant differences in FW between individuals with eSVD and the control group suggest that FW can be considered a marker for distinguishing subjects with and without early signs of cerebrovascular changes. This result, as well as our findings that individuals with eSVD showed lower levels of FA and higher levels of MD, is in line with previously reported results in genetic SVD [11]. Also, the elevated levels of RD compared to our healthy elderly control group were previously found by other authors [43]. In our study, however, no differences were observed for FW‐corrected parameters such as FA.t and MD.t. This contrasts with previous results by Duering and colleagues (2018) who found that FA.t and MD.t, even though weaker than the conventional DTI parameters FA and MD, allowed classification of genetically defined SVD patients from healthy controls [11]. This could possibly be attributed to the fact that Duering et al. (2018) investigated a different population by focusing on CADASIL (Cerebral Autosomal Dominant Arteriopathy with Subcortical Infarcts and Leukoencephalopathy; inherited cerebrovascular disease) patients. It might be reasonable to assume that CADASIL patients reflect a more severe population or that the authors studied the disease at a later stage, which would suggest that FW might be a more sensitive marker to identify differences in the early stage of SVD, while changes in the tissue‐related parameters may identify later stages where the deterioration also affected the brain tissue. Furthermore, the absence of differences in free water‐corrected DTI measures between healthy controls and individuals with early signs of SVD in this sample indicates that diffusion changes in SVD are primarily driven by extracellular fluid accumulation rather than disruptions in white matter fiber organization. This increase in free water may be attributed to several pathological processes such as a blood–brain barrier (BBB) disruption [44] or neuroinflammatory processes [45]. Therefore, one reason for increased free water could be that a disruption of the BBB leads to leakage of plasma components and water into the brain's extracellular space, resulting in increased free water values which can be detected through DTI [46]. Future studies combining DTI metrics with advanced imaging techniques for vascular and BBB assessment may provide a deeper understanding of these mechanisms.

Although these results are remarkable, the prognostic value of FW for neuropsychological assessments (such as memory, attention, or cognition in general) in patients with SVD remains unclear. As studies have shown that FW might represent a more specific indicator of cognitive decline compared to conventional diffusion MRI measures [47], FW indicates a relevant target for ongoing research on this topic.

Even though the correlation between age and FW as well as between age and FW‐uncorrected DTI measures was higher in magnitude for individuals with eSVD compared to healthy elderly, we did not observe any significant correlation differences between both groups after applying FDR correction. This suggests, for instance, that an increase in age is accompanied by an increase in extracellular FW, regardless of early cerebral pathology. We believe that this increase can be attributed to age‐related physiological changes such as alterations in brain tissue composition [48] (e.g., shrinkage of brain structures) which may lead to the expansion of the extracellular space and an increase in the proportion of free water. In addition, free water was found to be associated with cognitive decline, commonly observed in the aging population [49] which could explain the observed relationship with age even in individuals without eSVD.

Another fundamental result of our study was that the association between age and conventional DTI parameters was mediated by FW in NAWM. We therefore speculate that FW plays an important role in the relationship between age and all FW‐uncorrected DTI measures. This is also underpinned by the disappearing correlations between age and FW‐uncorrected DTI parameters after FW correction.

This study has some limitations as well. One constitutes its cross‐sectional study design, which prevents making statements about the long‐term prognostic value of FW. Further, while our results in general match current literature, future studies should consider the acquisition of multi‐shell diffusion data which may improve the accuracy of the FW model fit and may allow distinguishing further biological components that contribute to the increase in FW. Another limitation affects the choice of group sampling. The distinction between healthy elderly individuals and individuals with signs of SVD based on WMH, lacunes, and/or microbleeds could be considered arbitrary to this complex topic.

In conclusion, free water (FW) seems to be driving the association between age and FW‐uncorrected diffusion measures. It is remarkable that even in individuals with subtle and clinically silent cerebral white matter changes, noticeable increases in FW were observed. Consequently, FW might serve as a sensitive indicator of the earliest and clinically silent signs of small vessel disease (SVD) in healthy aging.

## Author Contributions

**Manuel Leitner:** methodology, writing – review and editing, writing – original draft, visualization, formal analysis, data curation, investigation, conceptualization. **Lukas Pirpamer:** methodology, writing – review and editing, supervision, formal analysis, data curation, software, investigation. **Edith Hofer:** methodology, writing – review and editing, supervision, data curation, formal analysis. **Stefan Ropele:** writing – review and editing, methodology, supervision, investigation. **Ofer Pasternak:** supervision, methodology, writing – review and editing, software, investigation. **Reinhold Schmidt:** conceptualization, writing – review and editing, resources, supervision, funding acquisition, project administration, investigation, methodology. **Marisa Koini:** project administration, conceptualization, methodology, writing – review and editing, funding acquisition, supervision, investigation, formal analysis.

## Ethics Statement

The study protocol was reviewed and approved by the ethics committee of the Medical University of Graz, Austria, and was conducted in accordance with the ethical guidelines of the Declaration of Helsinki. All subjects provided written informed consent to participate in this study. The participants were free to withdraw from the study at any time, without any disadvantage or consequences.

## Consent

All subjects provided written informed consent to participate in this study.

## Conflicts of Interest

The authors declare no conflicts of interest.

## Data Availability

The data used in the present study [50] is available at: https://doi.org/10.17632/j8bsyyxzvn.1.
